# Age-period-cohort analysis of dietary sodium, potassium, and sodium-to-potassium ratio in Korea

**DOI:** 10.4178/epih.e2025062

**Published:** 2025-11-04

**Authors:** Hee Ju Jun, Shieon Kim, Garam Jo

**Affiliations:** 1 Interdisciplinary Program in Precision Public Health, Graduate School of Korea University, Seoul, Korea; 2 Institute for Biomaterials, Korea University, Seoul, Korea

**Keywords:** Dietary sodium, Dietary potassium, Age factors, Birth cohort, Trend analysis, Hypertension

## Abstract

**OBJECTIVES:**

Excessive sodium and insufficient potassium consumption are major dietary contributors to hypertension (HTN) and cardiovascular diseases. The sodium-to-potassium ratio is a known predictor of blood pressure (BP) and HTN. This study evaluated 16-year-trends in dietary sodium, potassium, and the sodium-to-potassium ratio, and their associations with BP and HTN in Korea.

**METHODS:**

Data from 76,484 participants aged 19-79 years were obtained from the Korea National Health and Nutrition Examination Survey conducted in 2007-2022. Sodium and potassium intake were assessed using 24-hour recalls, and the sodium- to-potassium ratio was calculated. All values were energy-adjusted using the residual method. Age-period-cohort (APC) models were used to analyze temporal trends. Associations with BP and HTN were examined using multivariate linear and logistic regression models, adjusting for confounders.

**RESULTS:**

Energy-adjusted sodium intake decreased across all age groups, and potassium slightly increased in the recent 5 years, though both remained suboptimal relative to recommendations. APC analyses showed increasing potassium intake with age and a reversed U-shape cohort pattern. The sodium-to-potassium ratio decreased with age and calendar year but increased in recent cohorts. A higher sodium-to-potassium ratio was strongly associated with elevated systolic (β=0.028, p<0.001) and diastolic BP (β=0.036, p<0.001), and increased odds of HTN (odds ratio, 1.19; 95% confidence interval, 1.07 to 1.33). A linear association appeared when the sodium-to-potassium ratio exceeded 1.00 in the spline model.

**CONCLUSIONS:**

Despite improvements, sodium intake remains excessive, and potassium insufficient, particularly in younger adults and recent cohorts. Public health interventions should prioritize reducing sodium and promoting potassium-rich foods to reduce cardiovascular risk.

## GRAPHICAL ABSTRACT

[Fig f4-epih-47-e2025062]

## Key Message

Over the past 16 years, sodium intake among Korean adults has decreased but remains high, while potassium intake has recently increased yet still falls below recommended levels. Age-period-cohort (APC) analyses showed that the sodium-topotassium ratio has tended to increase in more recent cohorts. Because a higher sodium-to-potassium ratio is significantly associated with elevated blood pressure and a higher risk of hypertension, targeted public health efforts are needed.

## INTRODUCTION

Excessive sodium intake and insufficient potassium consumption are well-established contributors to hypertension (HTN) and cardiovascular diseases (CVDs), which remain leading causes of mortality worldwide [1]. Although the American Heart Association and the World Health Organization (WHO) recommend a low-sodium, high-potassium diet [2,3], most adults fail to meet these guidelines. In Korea, the 2020 Dietary Reference Intakes (KDRI) recommend 2,300 mg of sodium and 3,500 mg of potassium per day [4], yet actual intakes in 2022 were estimated at 2,600-3,800 mg of sodium and 2,300-3,000 mg of potassium [5]. This imbalance remains a major public health concern, especially in younger adults and recent cohorts, with the number of people diagnosed with HTN in Korea increasing from 3.0 million in 2002 to 11.5 million in 2022 [6].

Meanwhile, previous studies have reported the associations between dietary intake of sodium, potassium, or the sodium-to-potassium ratio, and blood pressure (BP) [7,8]. Of these, the sodium-to-potassium ratio reflecting the underlying physiological mechanisms of electrolyte balance has been proposed as a stronger predictor of the combined effects on vascular resistance and BP than either nutrient intake alone [9,10]. A systematic review of 41 studies identified the dietary sodium-to-potassium ratio as the most consistent predictor of BP [11]. Also, previous studies have reported that higher sodium-to-potassium ratios are significantly associated with HTN and stroke [6] and increased all-cause and CVD mortality [12-14], findings observed in cohorts from the United States [13] and Japan [12].

In Korea, higher sodium-to-potassium ratios have also been associated with higher BP and cardiovascular risk [15-17]. However, most prior studies were cross-sectional and thus could not quantify temporal changes or differences between generations. Moreover, sodium and potassium intake were often categorized or modeled linearly, limiting detection of potential non-linear dose-response associations across the full distribution of intake levels [18,19]. Many studies also focused on results from a single time point and thus could not distinguish long-term trends or birth-cohort differences and hence could not identify structural factors such as generational diet transitions or the impact of national nutrition policy.

Accordingly, in this study, we estimated population trends in sodium, potassium, and the sodium-to-potassium ratio and their associations with BP and HTN among Korean adults between 2007 and 2022, using the age-period-cohort (APC) model and restricted cubic spline (RCS) regression, respectively. The APC model disentangles the effects of age, survey period, and birth cohort, which is crucial to enable proper interpretation of temporal trends in dietary patterns observed in repeated cross-sectional surveys [20]. In addition, the RCS regression allows flexible modeling of non-linear dose-response relationships between continuous dietary intake and BP or HTN risk, providing better estimates without information loss from arbitrary categorization [21]. Through these methodological approaches, we addressed limitations of cross-sectional designs and systematically characterized temporal and generational patterns in sodium and potassium intake and their associations with cardiovascular health.

## MATERIALS AND METHODS

### Study population

This study used data from the Korea National Health and Nutrition Examination Survey (KNHANES) spanning from 2007 to 2022, established by the Korea Disease Control and Prevention Agency (KDCA). The KNHANES is an ongoing, cross-sectional survey that is nationally representative, providing information on the health and nutritional status of the Korean population. The survey employs a complex probabilistic sampling design with multiple stages of selection based on sex, age, and geographic regions. Additional details about the sampling designs for these surveys have been reported previously [22]. During the health examination, trained medical personnel performed anthropometric and biochemical measurements following standardized protocols.

The 2007-2022 KNHANES data included 95,228 participants aged 19-79 years. To reduce the risk of reverse causality from illness-related or pregnancy-related dietary changes [23,24], we excluded individuals with a history of cancer (n=3,481) or CVDs (n=2,073), females who were currently pregnant or lactating (n=453), or missing dietary data or reported implausible energy intake (<500 or >5,000 kcal/day; n=12,737), leaving a final of 76,484 participants (32,222 males and 44,262 females).

### Dietary intake and urinary excretion of sodium and potassium

The nutrition survey was performed by the KNHANES to identify dietary behavior, food, and nutrient intake [22]. Dietary sodium and potassium intakes were estimated using the one-day 24-hour dietary recall data in the KNHANES. Energy-adjusted sodium and potassium intakes were estimated using the residual method [25]. Dietary sodium-to-potassium ratio was calculated for each participant as the daily sodium intake divided by the daily potassium intake. Urinary sodium and potassium levels were measured by the ion-selective electrode method using a Hitachi Automatic Analyzer 7600-210 (Hitachi, Tokyo, Japan). We also calculated the urinary sodium-to-potassium ratio as the urinary excretion of sodium divided by the urinary potassium excretion to validate the association of the sodium-to-potassium ratio with BP and HTN. HTN was determined based on systolic blood pressure (SBP) ≥140 mmHg, diastolic blood pressure (DBP) ≥90 mmHg, or current use of antihypertensive medications.

### Covariates assessment

Covariates included demographic, lifestyle, and anthropometric factors. Age was recorded in years at the time of the survey. Sex was classified as males or females based on self-report. Education level was self-reported with four response options of elementary school graduate or below, middle school graduate, high school graduate, and university graduate or above. For analysis, these were combined into three categories of middle school graduate or below, high school graduate, and university graduate or above. Smoking status was self-reported and classified into three categories. Non-smokers were defined as participants who reported never smoking or who had smoked fewer than five packs of cigarettes in their lifetime. Past smokers were those who had smoked more than five packs in their lifetime but reported not currently smoking. Current smokers were those who had smoked more than five packs in their lifetime and reported currently smoking. Drinking status was categorized as non-drinkers, those who drank less than once per month, and those who drank once per month or more, based on self-reported frequency. Physical activity was assessed using a standardized questionnaire and expressed as metabolic equivalents (METs) [26]. Total energy intake (kcal/day) was calculated using 24-hour dietary recall data processed with a validated nutrient database. Body mass index (BMI) was computed as weight (kg) divided by height squared (m²), both measured during the health examination by trained personnel.

### Statistical analysis

We first analyzed the overall trends in energy-adjusted sodium and potassium intake individually, and the sodium-to-potassium ratio in 2007-2022, by sex and age groups. We calculated the age-standardized mean values for these dietary factors for each survey year, using the 2005 Korean Census population as the reference. To investigate the differences in age, period, and birth cohort effects concerning the following dietary sodium and potassium factors, we utilized APC models. The age effect refers to the physiological changes with aging. The period effect results from external events, such as war, economic crisis, and pandemic outbreak, that affect all age groups at a particular calendar time simultaneously, regardless of age and cohort. The cohort effect reflects differences in historical and social events based on the year of birth [27]. Age, period, and cohort are perfectly collinear in APC settings (i.e., cohort=calendar year-age), so fixed-effects models cannot uniquely attribute the overall linear trend to any single dimension. To deal with the identification problem, we adopted hierarchical APC cross-classified random effects models (HAPC-CCREM) [28]. In the HAPC-CCREM models, we used a mixed model framework, specifying age and its quadratic term as fixed effects at level 1, and periods and birth cohorts as random effects at level 2 [29]. This specification avoids arbitrary allocation of the linear drift and emphasizes inference on the dose-response relationship and on variance components attributable to period and cohort. This study focused on adults aged 19 years to 79 years, centering age around the population mean of 49 years. The period variable indicated 1-year intervals from 2007 to 2022, while birth cohorts were grouped into 5-year intervals, except for extreme cohorts with small numbers, ranging from the 1928-1939 cohort to the 1990-2003 cohort. Data were analyzed, accounting for survey sample weights. Using the APC models, we estimated the mean intakes of sodium, potassium, and the sodium-to-potassium ratio for each age, period, and birth cohort.

The associations of dietary sodium, potassium, and their ratio with BP levels were analyzed using multiple linear regression analysis. Multivariable model was adjusted for age, sex (males or females), education (middle school graduate and below, high school graduate, or university graduate and above), smoking status (non-smokers, past smokers, or current smokers), drinking status (non-drinkers, less than once per month, or once per month or more), exercise assessed as METs, total energy intake (kcal/day), and BMI. For a categorical variable, dietary sodium intake was divided into three groups based on the 2020 KDRI: <1,500 mg/day, 1,500-2,300 mg/day, and ≥2,300 mg/day, with 1,500-2,300 mg/day serving as the reference group, while other variables were divided into quartiles, using the lowest quartile as the reference category. Multiple logistic regression models were used to estimate odds ratios (ORs) and 95% confidence intervals (CIs) of the association between dietary factors and HTN. In addition, multivariable logistic regression models with RCS functions using 3 knots at the 10th percentile, 50th percentile, and 90th percentile were applied to examine a possible dose-response association of individual dietary sodium and potassium intakes and their ratio with HTN, adjusting for covariates. We used multivariable logistic regression with RCS to model dose-response relationships in continuous dietary factors. This approach can flexibly capture non-linearity, estimate nadir intake and inflection points, and visualize the full curve, while minimizing information loss from arbitrary categorization and offering greater robustness than simple polynomials. It thereby identifies intake levels associated with the lowest odds of HTN across dietary factors [21].

A two-sided p-value of <0.05 was considered significant. All analyses were performed using SAS version 9.4 (SAS Institute Inc., Cary, USA) and Stata/MP version 18.0 (StataCorp., College Station, USA).

### Ethics statement

All participants provided written informed consent, and the Institutional Review Board of the KDCA approved all procedures (IRB No. 2007-02CON-04-P, 2008-04EXP-01-C, 2009-01CON-03-2C, 2010-02CON-21-C, 2011-02CON-06-C, 2012-01EXP-01-2C, 2013-07CON-03-4C, 2013-12EXP-03-5C, 2018-01-03-P-A, 2018-01-03-C-A, 2018-01-03-2C-A, 2018-01-03-5C-A, and 2018-01-03-4C-A).

## RESULTS

### Trends in dietary sodium and potassium intake

**Figure 1.**
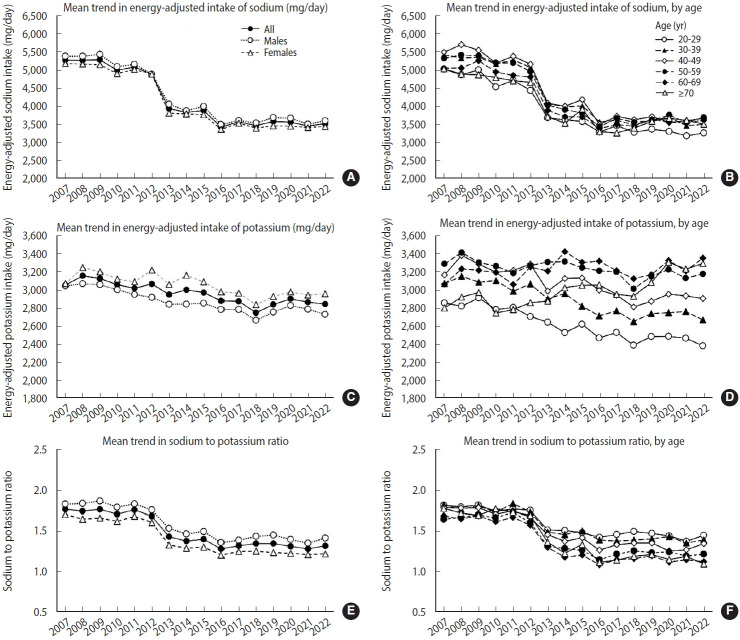
Mean trends in energy-adjusted dietary sodium and potassium intake from 2007 to 2022. The trends in dietary sodium intake (mg/day) are shown (A) by sex and (B) by age group, and trends in dietary potassium intake (mg/day) are shown (C) by sex and (D) by age group, all energy-adjusted using the residual method. Mean trends in the dietary sodium-to-potassium ratio are presented (E) by sex and (F) by age group.

We examined trends in mean dietary sodium and potassium intake from 2007 to 2022, and the results are presented in Supplementary Material 1. During this period, mean dietary sodium intake showed a consistent downward trend, decreasing from 4,854 mg/day in 2007 to 3,215 mg/day in 2022 (Supplementary Material 1A). To account for total energy intake, we investigated trends in energy-adjusted sodium and potassium intake, and these results are illustrated in Figure 1. Though this downward trend was also observed for energy-adjusted sodium intake, it plateaued after 2016 (Figure 1A). This pattern was similar in both males and females, and males consistently consumed more sodium throughout the entire study period, even after energy adjustment (3,605 mg/day for males vs. 3,445 mg/day for females in 2022). When it comes to age-specific patterns, mean sodium intake decreased consistently across all age groups, though the slope of decline varied (Supplementary Material 1B). However, after energy adjustment, the gaps between age groups were attenuated. In recent years, from 2018 to 2022, energy-adjusted sodium intake was lowest among the younger population aged 19-29 years (Figure 1B).

Over the 16-year study period, mean dietary potassium intake demonstrated an overall decreasing trend, declining from 2,876 mg/day in 2007 to 2,604 mg/day in 2022 (Supplementary Material 1C). In contrast, energy-adjusted potassium intake showed a marginal upward trend after 2018 (Figure 1C). Throughout the entire study period, energy-adjusted potassium intake was lower in males compared to females. Energy-adjusted potassium intake patterns varied by age groups, showing that younger groups aged 19-39 years had slightly lower potassium consumption and older groups aged 60 years or more maintained an upward trend over time (Figure 1D).

### Sodium-to-potassium ratio trends

The sodium-to-potassium ratio significantly decreased over the study period (Figure 1E), reflecting modest improvements. However, the sodium-to-potassium ratio remained higher than the optimal value of approximately 1.00, ranging from 1.32 to 1.77. The sodium-to-potassium ratio declined across all age groups (Figure 1F). Particularly, age-specific differences became more pronounced, with this gap widening since 2012.

### Age-period-cohort effects

**Figure 2.**
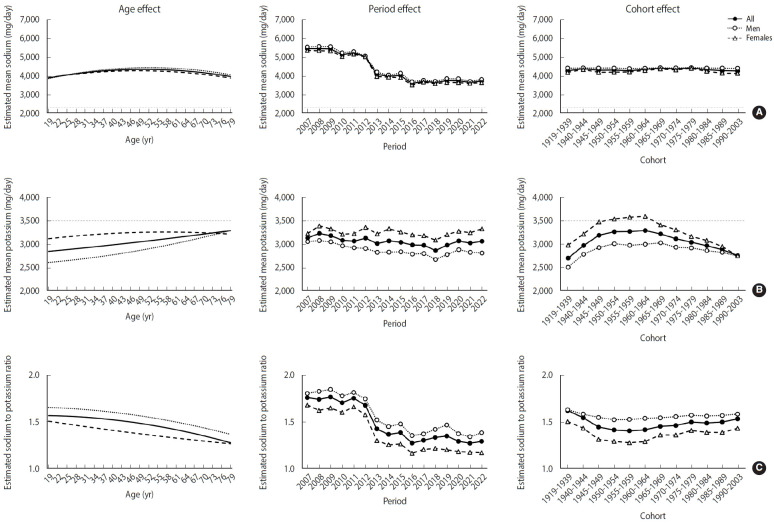
Age–period–cohort analysis of energy-adjusted dietary sodium, potassium, and sodium-to-potassium ratio. Mean energy-adjusted (A) sodium, (B) potassium, and (C) sodium-to-potassium ratio were estimated using hierarchical age–period–cohort cross-classified random-effects models from 2007 to 2022. Energy adjustment was performed using the residual method. Horizontal dashed lines indicate the 2020 Dietary Reference Intakes for Koreans: the chronic disease risk reduction intake for sodium (2,300 mg/day) and the adequate intake for potassium (3,500 mg/day).

The APC analyses were performed to independently detect age, period, and birth cohort effects on dietary sodium, potassium, and their ratio. Figure 2 illustrates the estimated age, period, and cohort effects on dietary sodium, potassium and their ratio. Before examining their effects, we first evaluated the goodness-of-fit of different model structures, including the AC (age–cohort), AP (age–period), PC (period–cohort), APC, and age-only models and Supplementary Material 2 summarized the goodness-of-fit statistics for these models. The APC model generally provided the best explanation of variation, while the AP model for energy-adjusted sodium and the AC model for energy-adjusted potassium showed comparable or slightly better performance. For the sodium-to-potassium ratio, the APC model achieved the lowest Akaike information criterion and Bayesian information criterion and the smallest residual deviance (Supplementary Material 2), outperforming the AP, AC, and PC specifications. This indicates that age, period, and cohort effects jointly improve model fit, reinforcing the need to account for all three components to fully capture temporal trends and avoid oversimplified interpretations. Based on these model comparisons, we then examined the independent age, period, and cohort effects in detail. For dietary sodium (Figure 2A), a significant period effect was observed, with a consistent downward trend over time. However, there was no clear pattern across birth cohorts, suggesting that sodium intake levels remained stable across different birth cohorts when accounting for age and calendar time considered. For dietary potassium (Figure 2B), the age effect showed an upward trend with age. Period effects indicated a gradual decrease in potassium intake through 2018, with a slight increase observed thereafter. The cohort effect showed a reversed U-shape pattern, peaking at 3,250 mg/day among those born in 1960-1964, and then gradually decreasing with more recent birth cohorts. On the other hand, the sodium-to-potassium ratio decreased steadily with age (Figure 2C). The cohort effect decreased until the 1950s birth cohort (estimated mean: 1.41) and increased thereafter. Across all cohorts, males had higher sodium-to-potassium ratios than females, primarily due to lower potassium intake.

#### Associations of dietary sodium and potassium intake and their ratio with blood pressure and hypertension

Associations of dietary sodium intake, potassium intake, and their ratio with BP and HTN are presented in Table 1. Higher sodium intake was linearly associated with increased DBP (standardized β: 0.038 per unit increase, p<0.001), while higher potassium intake was inversely associated with SBP (standardized β: -0.048 per unit increase, p<0.001). Those who consumed less than 1,500 mg/day of sodium intake had increased SBP compared to those with optimal levels of 1,500-2,300 mg/day (standardized β: 0.010 per unit increase, p=0.020). An inverse association was observed between dietary potassium intake and HTN (OR, 0.82; 95% CI, 0.76 to 0.89) when comparing the highest quartile (Q4) and lowest quartile (Q1) intake groups. Moreover, higher sodium-to-potassium ratios were significantly associated with increased SBP (standardized β: 0.028 per unit increase, p<0.001), DBP (standardized β: 0.036 per unit increase, p<0.001), and HTN outcomes (OR, 1.19; 95% CI, 1.07 to 1.33). Consistent with the dietary sodium-to-potassium ratio, the urinary ratio was also significantly associated with SBP (standardized β: 0.074 per unit increase, p<0.001), DBP (standardized β: 0.047 per unit increase, p<0.001), and HTN (OR, 1.06; 95% CI, 1.03 to 1.08).

### Dose-response associations of dietary sodium and potassium intake and their ratio with hypertension

**Figure 3.**
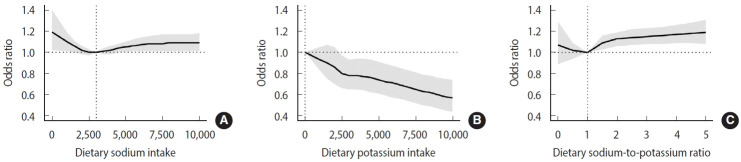
Dose–response associations of dietary sodium intake (A), potassium intake (B), and the sodium-to-potassium ratio (C) with hypertension. Thick lines represent odds ratio estimates, with gray-shaded areas indicate 95% confidence intervals. Reference values were set at 3,000 mg/day for dietary sodium intake, 0 mg/day for dietary potassium intake, and 1.0 for the sodium-to-potassium ratio. All analyses were adjusted for age, sex, education level, smoking status, drinking status, physical activity, total energy intake, and body mass index.

We used a RCS model to examine the associations of dietary sodium, potassium and their ratio with HTN, and the results are presented in Figure 3. A curvilinear association was observed between dietary sodium intake and HTN, with the lowest odds at around 3,000 mg/day. Interestingly, sodium intake below 1,500 mg/day (OR, 1.06; 95% CI, 1.00 to 1.11) and above 4,500 mg/day (OR, 1.04; 95% CI, 1.00 to 1.08) were both significantly associated with increased odds of HTN. In comparison, dietary potassium intake showed a linear inverse association with HTN risk, with significantly lower odds observed at intakes of 2,500 mg/day or higher (OR, 0.80; 95% CI, 0.66 to 0.97). Based on the spline analysis, the ORs for HTN increased linearly when the sodium-to-potassium ratio exceeded 1.00.

## DISCUSSION

In this study, we examined long-term trends in dietary sodium, potassium, and the sodium-to-potassium ratio among Korean adults between 2007 and 2022, and their associations with BP and HTN. Over this period, energy-adjusted sodium intake and the sodium-to-potassium ratio declined, and potassium intake showed modest gains. Yet sodium intake remained above, and potassium intake below, recommended levels [4]. Thus, the sodium-to-potassium ratio remained consistently above the optimal value of about 1.0, with the most unfavorable balance in younger adults and in males. Because this ratio was strongly and consistently associated with BP and HTN, it may serve as a more meaningful marker of dietary quality than sodium or potassium alone. These findings align with earlier national reports in Korea [30] and with international patterns, such as persistently high sodium intakes in the United States [31] and severe imbalance in China [7], highlighting the ongoing global challenge of sodium-potassium despite health initiatives.

The APC analysis added further perspective. The decline in sodium intake was largely explained by period effects, aligning with the timing of national sodium reduction policies launched in 2012 [32]. These initiatives, which include education, healthier food environments, and reformulation of processed foods, produced measurable reductions, with national targets even reached ahead of schedule [32,33]. By contrast, potassium intake was shaped more strongly by cohort effects. Intakes peaked among those born in the 1960s and declined across more recent cohorts, suggesting generational shifts toward higher consumption of ultra-processed foods and sugar-sweetened beverages and reduced fruit and vegetable intake [34-37]. This dietary pattern has been associated with adverse cardiometabolic health outcomes in Korean populations [38], and it has also been reported to increase BP and cardiovascular risk through an elevated inflammatory dietary index [39]. Sex differences were also clear, with males consistently consuming more sodium and less potassium than females. Taken together, these findings suggest that while sodium-focused policies have had a broad impact, improving potassium intake will require strategies tailored to cohort and sex, with particular attention to younger males.

Previous studies that have analyzed the association between sodium intake and CVD risk have reported mixed results, including strong positive correlations, U-shaped curves, and even null results [36]. This lack of consistency suggests the possibility of complex interactions between sodium and potassium intake. Consistent with these findings, our study also demonstrated a non-linear, U-shaped association between sodium intake and the odds of HTN, as well as a linear negative association for potassium intake. Remarkably, we observed that both low (<1,500 mg/day) and high (>4,500 mg/day) sodium intake were associated with an increased risk of HTN, whereas high potassium intake (≥2,500 mg/day) was associated with a significantly lower risk of HTN. Moreover, the spline analysis in this study showed a linear increase in the ORs for HTN when the sodium-to-potassium ratio exceeded 1.0, supporting the WHO recommendation to maintain the ratio below or around 1.0 for cardiovascular health [3]. Numerous studies have demonstrated strong associations between both dietary and urinary sodium-to-potassium ratios and the risk of CVD, HTN, ischemic strokes, and chronic kidney disease [12,37,40]. A meta-analysis combining randomized controlled trials found that a lower urinary sodium-to-potassium ratio was associated with a significant reduction in SBP and DBP by -1.09 mmHg and -1.42 mmHg, respectively [41]. In addition, an analysis combining six large cohorts found that a high sodium-to-potassium ratio, measured using multiple 24-hour urine samples, was associated with a 62% higher risk of CVD [42]. The MESA (Multi-Ethnic Study of Atherosclerosis) study reported a 1.5-fold higher risk of stroke in participants with a urinary sodium-to-potassium ratio greater than 1.0, compared to those below 1.0 [43]. The Nagahama study in Japan also showed that the urinary sodium-to-potassium ratio independently predicted BP, regardless of absolute electrolyte excretion [44]. Our findings, along with previous studies, suggest that the sodium-to-potassium ratio may be a more accurate predictor of CVD risk than sodium or potassium intake alone, further supporting its clinical value.

The biological basis for these associations is well established. High sodium intake increases renal sodium reabsorption, suppresses the renin-angiotensin-aldosterone system [45], and enhances sympathetic activity [46], while potassium promotes sodium excretion, reduces renin secretion, and induces vasodilation [47]. A combined state of high sodium and low potassium worsens sodium retention and potassium depletion through renal transporter activation [48]. These mechanisms explain why the sodium-to-potassium ratio repeatedly emerged as the most sensitive and integrative indicator of BP regulation and cardiovascular risk.

This study has several strengths. First, we used nationally representative data of the Korean civilian population, enhancing the generalizability of the findings. Second, the APC model was applied to evaluate age, period, and birth cohort effects on sodium and potassium intake, also helping to identify the potential impact of national dietary initiatives. Third, sodium, potassium, and their ratio were analyzed together, providing a more physiologically relevant measure. This study also has some limitations. First, the use of single-day 24-hour dietary recall data may introduce recall bias and misclassification and may not adequately reflect usual intake due to seasonal variation. Second, while the APC model could demonstrate the independent effects of age, period, and cohort on sodium and potassium intake trends, it does not directly explain the underlying causes of these changes. However, by comparing the timing of observed period effects with national dietary initiatives, we were able to infer potential explanations. Third, A substantial number of participants (n=12,737) were excluded due to implausible or missing dietary data. The excluded group had significantly more males and younger individuals, raising the possibility of selection bias. There were no significant differences across survey years, except for 2020, when only 166 of 192 primary sampling units (86.4%) were surveyed due to the COVID-19 (coronavirus disease 2019) pandemic [49]. These factors should be considered when interpreting the results. Lastly, due to its cross-sectional design, causality cannot be established, and age effects may be influenced by survivor selection. We therefore interpret the age effects cautiously and place more emphasis on the period effects, which are less sensitive to survival bias. To reduce bias, we excluded participants with prior CVD or cancer, adjusted for lifestyle factors and BMI. Although we applied age-standardization and corroborated patterns using urinary sodium-to-potassium ratio, residual selection and reverse causation cannot be ruled out. Further studies using longitudinal designs and including multiple ethnic groups are required to clarify the effects of sodium and potassium intake, and their ratio, on the development of HTN and to better understand their associations.

In conclusion, despite progress in lowering sodium intake, the sodium-to-potassium ratio remains elevated in Korea, particularly among younger cohorts. These findings highlight the importance of complementing sodium reduction policies with active promotion of potassium intake, for instance, through subsidies for fruits and vegetables, clearer front-of-pack labeling, and targeted efforts aimed at younger males. Implementing an integrated approach that combines tailored strategies to reduce sodium and increase potassium, while considering population characteristics and living environments [50], will be key to effectively reducing the burden of HTN and CVD in Korea.

## Figures and Tables

**Figure f4-epih-47-e2025062:**
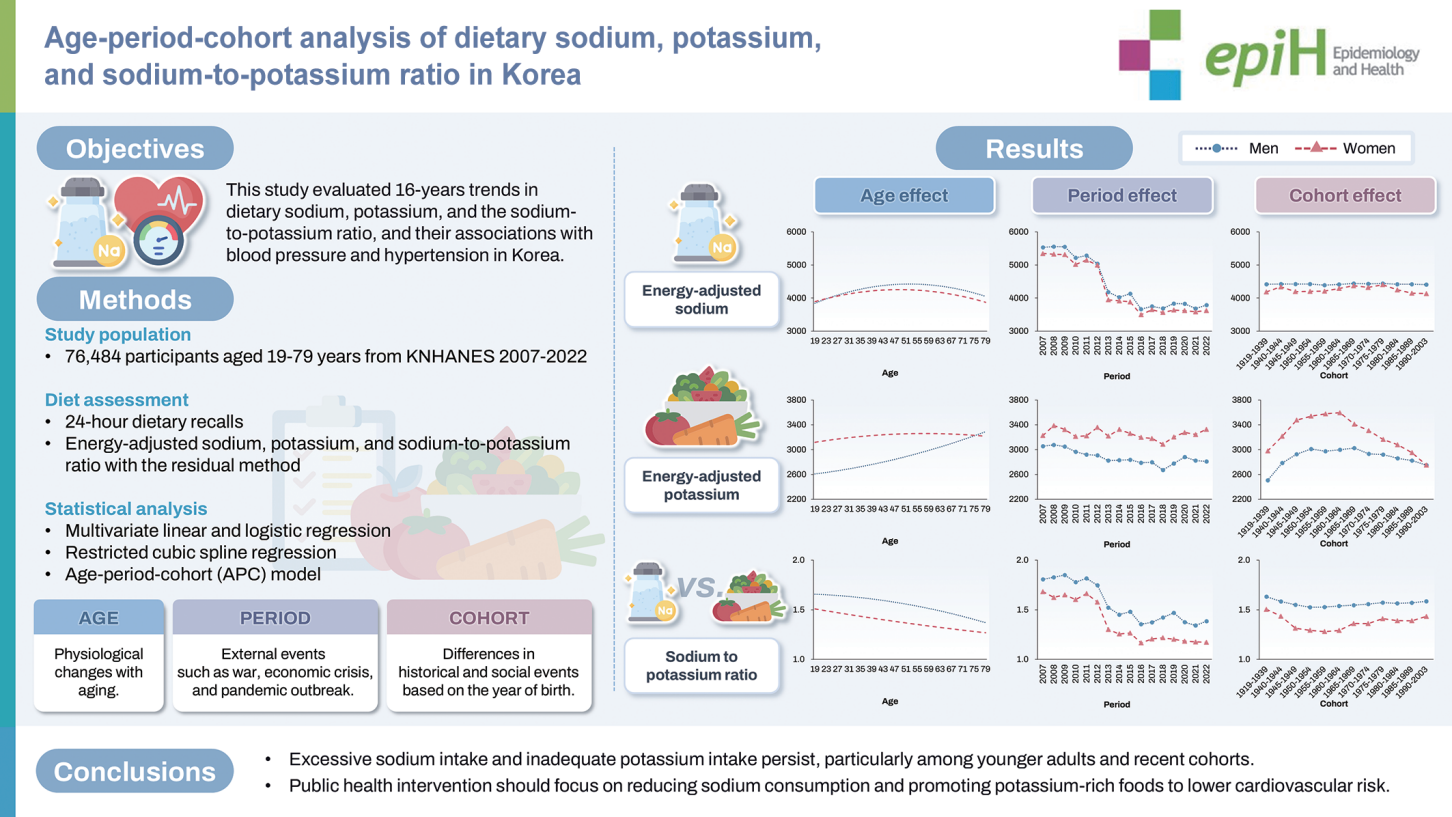


**Table 1.** Association of sodium, potassium intake, and dietary and urinary sodium-to-potassium ratio with blood pressure and HTN^1^

Variables		SBP		DBP		HTN	
		β (95% CI)	p-value	β (95% CI)	p-value	OR (95% CI)	p-value
Dietary sodium intake (mg)	Cont.	0.003 (-0.000, 0.000)	0.462	0.038 (0.000, 0.000)	<0.001	1.00 (1.00, 1.00)	0.017
	<1,500	0.010 (0.087, 1.042)	0.020	0.001 (-0.276, 0.369)	0.778	1.02 (0.94, 1.11)	0.600
	1,500-2,300	Reference		Reference		1.00 (reference)	
	>2,300	-0.004 (-0.497, 0.194)	0.390	0.023 (0.305, 0.773)	<0.001	0.97 (0.92, 1.04)	0.423
Dietary potassium intake	Cont.	-0.048 (-0.001, -0.000)	<0.001	-0.007 (-0.000, 0.000)	0.197	0.99 (0.99, 0.99)	<0.001
	Q1	Reference		Reference		1.00 (reference)	
	Q2	-0.030 (-1.525, -0.787)	<0.001	-0.003 (-0.334, 0.166)	0.510	0.91 (0.85, 0.97)	0.005
	Q3	-0.043 (-1.988, -1.220)	<0.001	0.001 (-0.234, 0.286)	0.844	0.87 (0.81, 0.93)	<0.001
	Q4	-0.065 (-2.821, -1.932)	<0.001	-0.004 (-0.380, 0.204)	0.526	0.82 (0.76, 0.89)	<0.001
Dietary sodium to potassium ratio	Cont.	0.028 (0.460, 0.767)	<0.001	0.036 (0.383, 0.591)	<0.001	1.08 (1.05, 1.11)	<0.001
	Q1	Reference		Reference		1.00 (reference)	
	Q2	0.013 (0.171, 0.858)	0.003	0.011 (0.031, 0.495)	0.026	1.05 (0.98, 1.11)	0.154
	Q3	0.013 (0.148, 0.832)	0.004	0.025 (0.360, 0.823)	<0.001	1.10 (1.04, 1.17)	0.002
	Q4	0.035 (0.951, 1.630)	<0.001	0.044 (0.777, 1.237)	<0.001	1.17 (1.10, 1.24)	<0.001
Urinary sodium to potassium ratio	Cont.	0.074 (0.519, 0.754)	<0.001	0.047 (0.172, 0.332)	<0.001	1.06 (1.03, 1.08)	<0.001
	Q1	Reference		Reference		1.00 (reference)	
	Q2	0.034 (0.643, 1.854)	<0.001	0.001 (-0.400, 0.425)	0.952	0.97 (0.86, 1.09)	0.578
	Q3	0.045 (1.448, 2.668)	<0.001	0.024 (0.142, 0.974)	0.009	1.00 (0.89, 1.12)	0.980
	Q4	0.089 (2.705, 3.940)	<0.001	0.047 (0.676, 1.518)	<0.001	1.25 (1.12, 1.40)	<0.001

HTN, hypertension; SBP, systolic blood pressure; DBP, diastolic blood pressure; OR, odds ratio; CI, confidence intervals; Cont., continuous; Q, quartile.

1 Adjusted associations were estimated using multiple linear, logistic, and Cox regression models; Risk estimates were calculated by comparing each quartile to quartile 1 (reference group), adjusting for age, sex, education, smoking status, drinking status, physical activity, total energy intake, and body mass index.

## Supplementary materials

Supplementary Material 1. Mean trends in dietary sodium and potassium intake in 2007–2022.

Supplementary Material 2. Goodness of fit of APC model for energy-adjusted sodium and potassium and sodium-to-potassium ratio
