# Supplementary material for: Age-period-cohort analysis of dietary sodium, potassium, and sodium-to-potassium ratio in Korea
Source: Epidemiol Health. 2025 Nov 4;47:e2025062. doi: 10.4178/epih.e2025062 (PMC12884018; doi:10.4178/epih.e2025062)
Supplement: Supplementary Material 1. — Mean trends in dietary sodium and potassium intake in 2007–2022. [file epih-47-e2025062-Supplementary-1.docx]

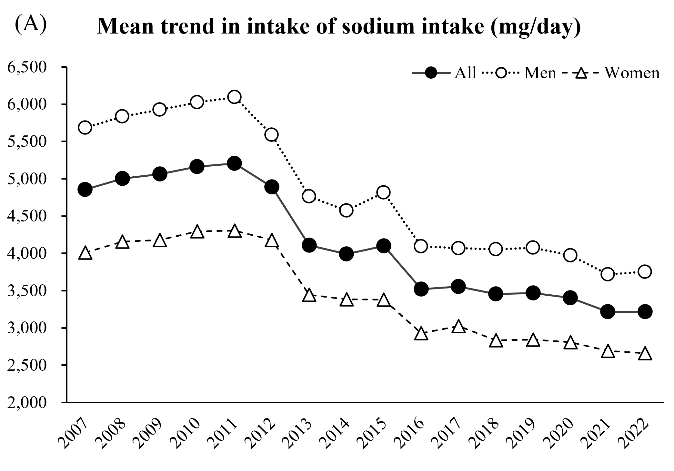

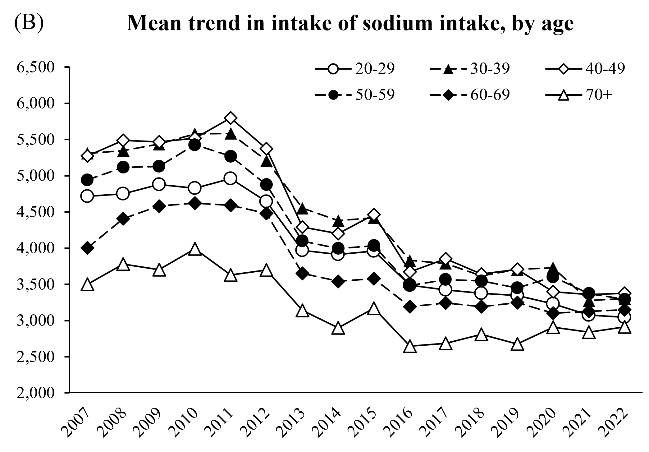

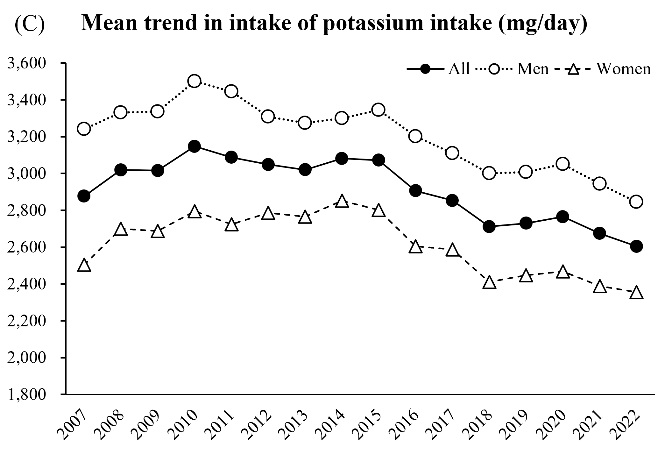

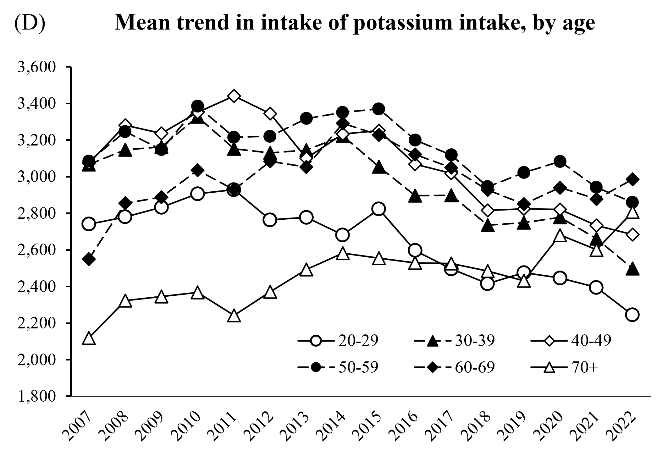


**Supplementary Material 1. Mean trends in dietary sodium and potassium intake in 2007–2022.** The trends in (A) dietary sodium intake (mg/day) and (C) potassium intake (mg/day) (B) Mean daily sodium intake (mg/day) by age groups (D) Mean daily sodium intake (mg/day) by age groups
