# Supplementary material for: Age-period-cohort analysis of dietary sodium, potassium, and sodium-to-potassium ratio in Korea
Source: Epidemiol Health. 2025 Nov 4;47:e2025062. doi: 10.4178/epih.e2025062 (PMC12884018; doi:10.4178/epih.e2025062)
Supplement: Supplementary Material 2. — Goodness of fit of APC model for energy-adjusted sodium and potassium and sodium-to-potassium ratio [file epih-47-e2025062-Supplementary-2.docx]

| **Supplementary Material 2. Goodness of fit of APC model for energy-adjusted sodium and potassium and sodium-to-potassium ratio** | | | | | | |
| --- | --- | --- | --- | --- | --- | --- |
| **Index** | **Model** | **AIC** | **DEV** | **df** | **Δ DEV** | **Δ df** |
| Energy-adjusted sodium | APC | 1,402,345 | 1,402,333 | 6 | 0 | 0 |
|  | AC | 1,405,035 | 1,405,031 | 5 | 2,698 | -1 |
|  | AP | 1,402,329 | 1,402,323 | 5 | -10 | -1 |
|  | PC | 1,402,410 | 1,402,404 | 4 | 71 | -2 |
|  | Age | 1,411,236 | 1,411,234 | 4 | 8,901 | -2 |
| Energy-adjusted potassium | APC | 1,297,032 | 1,297,020 | 6 | 0 | 0 |
|  | AC | 1,297,018 | 1,297,012 | 5 | -8 | -1 |
|  | AP | 1,297,633 | 1,297,629 | 5 | 609 | -1 |
|  | PC | 1,297,046 | 1,297,040 | 4 | 20 | -2 |
|  | Age | 1,298,326 | 1,298,324 | 4 | 1,304 | -2 |
| Sodium-to-potassium ratio | APC | 186,397 | 186,385 | 6 | 0 | 0 |
|  | AC | 187,997 | 187,993 | 5 | 1,608 | -1 |
|  | AP | 186,582 | 186,578 | 5 | 193 | -1 |
|  | PC | 186,442 | 186,436 | 4 | 51 | -2 |
|  | Age | 191,772 | 191,770 | 4 | 5,385 | -2 |
| AC, age–cohort model; AIC, Akaike information criteria; AP, age–period model; APC, age–period–cohort model; DEV, deviance; df, degree of freedom; PC, period–cohort model | | | | | | |
